# Possible functional role of olfactory subsystems in monitoring inhalation and exhalation

**DOI:** 10.3389/fnana.2014.00107

**Published:** 2014-09-29

**Authors:** Kensaku Mori, Hiroyuki Manabe, Kimiya Narikiyo

**Affiliations:** ^1^Department of Physiology, Graduate School of Medicine, The University of TokyoTokyo, Japan; ^2^CREST, Japan Science and Technology AgencyTokyo, Japan

**Keywords:** respiration phase, Grueneberg ganglion, CO_2_-detection, cool sensor, necklace glomeruli

In parallel with progress in understanding the canonical main olfactory system, a recent advance has accelerated elucidation of the structural organization and functional properties of the olfactory subsystems. These include the ganglion of Grueneberg, septal organ, and specific subsets of olfactory sensory neurons in the main olfactory epithelium. The emerging concept of the olfactory subsystems is that each subsystem expresses distinct classes of chemoreceptors and signal transduction molecules, and thereby detects distinct categories of odor or pheromone molecules. The chemical signals detected by the subsystems are sent via their axons to subsystem-specific domains in the glomerular map of the main olfactory bulb (Mori and Sakano, 2011).

For example, a small subset of olfactory sensory neurons in the main olfactory epithelium of mice expresses a second class of odorant receptors, trace amine-associated receptors (TAARs) (Liberles and Buck, 2006), and detects amine compounds (Zhang et al., 2013) that are released from male urine, predators, spoiled foods or dead animals. TAAR-expressing olfactory sensory neurons project axons to glomeruli clustered in a specific region in the dorsal domain of the glomerular map (Johnson et al., 2012; Pacifico et al., 2012). Another example is a subset of neurons in the Grueneberg ganglion that detects an alarm pheromone, 2-*sec*-butyl-4, 5-dihydrothiazole (SBT), which is released from conspecific mice under a threatening situation (Brechbuhl et al., 2013). This subset of Grueneberg ganglion neurons mediates the alarm pheromone-induced innate fear response and sends axons to the glomeruli at the dorso-posterior part of the main olfactory bulb.

In this opinion article, we argue for the notion that on top of their major role as detectors of distinct categories of chemical signals, some of the olfactory subsystems play important roles in detecting the timing of the inhalation phase and exhalation phase of respiration. Olfactory perception occurs in discrete breaths (sniffs). Therefore, respiration rhythm plays a key role orchestrating the information processing mode across a number of regions in the central olfactory system, which includes the olfactory bulb and numerous areas of the olfactory cortex (Mori and Manabe, 2014).

Each respiration cycle consists of an inhalation phase followed by an exhalation phase (Figure 1). During the inhalation phase, odorants in the external world are drawn, together with external cool air, into the nasal cavity and thereby activate olfactory sensory neurons in the olfactory epithelium. Therefore, the central olfactory system is engaged in the processing of olfactory sensory inputs from the external world during the inhalation phase. During the exhalation phase, warm lung air flows through the pharynx into the nasal cavity via the retronasal pathway. The air stream then flows outwardly through the nostrils, preventing external odors from reaching the nasal cavity. The central olfactory system is thus temporarily isolated from the external odor world, and typically process olfactory information off-line during the exhalation phase. However, a unique mode of olfactory sensory neuron stimulation occurs during eating: the central olfactory system receives retronasal odor stimulation from foods in the mouth during the exhalation phase (Gautam and Verhagen, 2012). Thus, in awake behaving states, the central olfactory system appears subject to respiration phase-paced changes in information processing mode. In agreement with this idea, a recent study showed that mice can behaviorally discriminate the sniff phase of optogenetically driven activation of olfactory sensory neurons (Smear et al., 2011).

**Figure 1:**
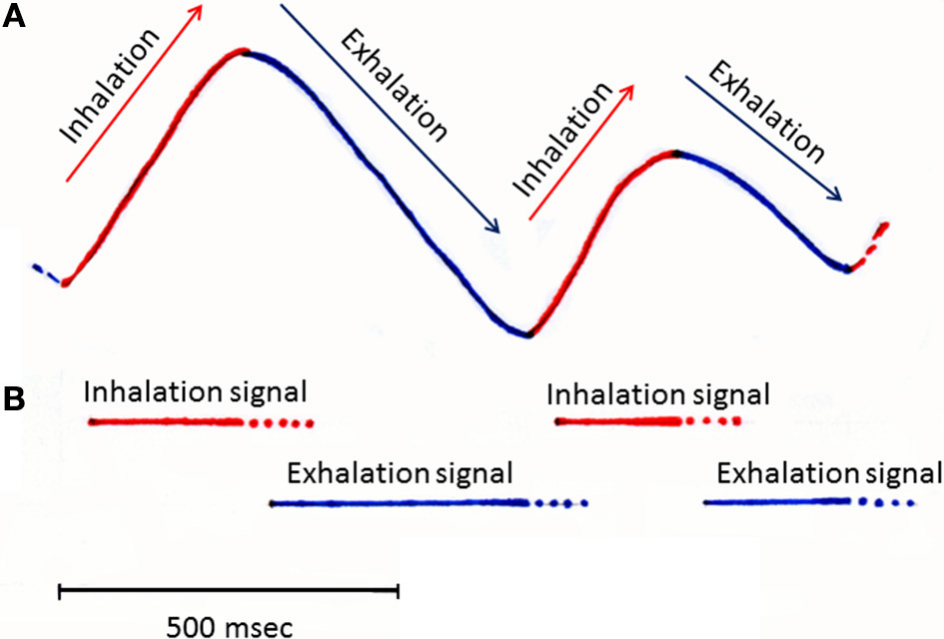
**A schematic diagram illustrating the respiration rhythms and possible timing of inhalation and exhalation signals**. Inhalation and exhalation phases of slow respirations recorded from a rat during awake resting. Upper swing of trace **(A)** (output from a thermocouple placed in the nasal cavity) indicates the inhalation phase (shown red), while a downswing indicates the exhalation phase (shown blue). Bars in **(B)** indicate the possible timing of inhalation (red bars) and exhalation signals (blue bars) which might be detected by specific subsets of olfactory sensory neurons and sent to the olfactory bulb.

We speculate that in order for the central olfactory system to perform respiration phase-specific changes in the mode of information processing, it should receive signals regarding the timing of inhalation and exhalation. Here, we hypothesize that some of the olfactory subsystems function as monitors of the inhalation and exhalation phases of respiration. Specifically, we propose that these olfactory subsystems convey information on dynamic change in the direction of respiratory air flow to the central olfactory system.

The Grueneberg ganglion is an olfactory subsystem located at the entrance of the nose, a strategically important place for immediately detecting the inhalation of external chemical cues and air. As stated above, a subset of sensory neurons in the ganglion respond to alarm pheromone SBT (Brechbuhl et al., 2013), while other subsets of ganglion neurons respond to other chemical cues. Interestingly, Grueneberg ganglion cells express thermosensitive potassium channel TREK-1 (Stebe et al., 2014) and respond to cool ambient temperature (Mamasuew et al., 2008). Because an inhalation of external cool air is a good stimulus for these sensory neurons, we speculate that the additional function of Grueneberg ganglion neurons is to monitor the timing of inhalation, especially when mice sniff in a cool environment. At relatively warm environmental temperature, however, Grueneberg ganglion neurons may not be a reliable monitor of inhalation (Mamasuew et al., 2008), and hitherto unknown olfactory or trigeminal subsystems might contribute to signal the inhalation phase.

Axons of Grueneberg ganglion cells project to glomeruli at the dorso-posterior part of the main olfactory bulb, forming a semicircle around the anterior part of the accessory olfactory bulb (Fuss et al., 2005; Koos and Fraser, 2005; Fleischer et al., 2006; Roppolo et al., 2006; Storan and Key, 2006). It should be noted that these glomeruli are distinct from guanylyl cyclase-D (GC-D)-positive necklace glomeruli, which receive axons from CO_2_-sensitive olfactory sensory neurons in the main olfactory epithelium (Fuss et al., 2005).

A small subset of olfactory sensory neurons in the olfactory epithelium express GC-D and cAMP-stimulated phosphodiesterase-2A (PDE2A). Because these olfactory sensory neurons project axons to the necklace glomeruli located at the posterior-most part of the glomerular map, they are called necklace olfactory neurons (Luo, 2008). These necklace olfactory neurons detect atmospheric carbon dioxide (CO_2_) with high sensitivity (Hu et al., 2007). We speculate that during the exhalation phase, the CO_2_-sensitive necklace olfactory neurons are exposed to and activated by the air from the lung, which contains a high concentration of CO_2_. We therefore hypothesize that the necklace glomerular system may have an additional functional role, namely monitoring the exhalation phase of respiration. However, the CO_2_ levels will not be raised in the anatomical dead space air in which gas exchange has not occurred. Therefore, the CO_2_-sensitive necklace olfactory neurons may not reliably signal the start of exhalation and may signal the latter phase of exhalation. It is possible that hitherto unknown olfactory or trigeminal subsystems also might contribute the detection of the exhalation phase.

The septal organ is an island of olfactory epithelium located at the ventral base of the nasal septum. About 70% of olfactory sensory neurons in the septal organ respond to both odor stimulation and to mechanical stimulation, suggesting that they respond with spike discharges in synchrony with the nasal air flow even without odor input (Grosmaitre et al., 2007). The olfactory sensory neurons in the septal organ project axons to a subset of glomeruli in the medio-ventral part of the posterior olfactory bulb (Ma et al., 2003). In addition, about 50% of olfactory sensory neurons show mechanosensitivity in the main olfactory epithelium. On these bases, the mechanosensitive neurons in the septal organ and main olfactory epithelium may respond to air flow in the nasal cavity during both inhalation and exhalation, and drive synchronization of the activity of olfactory bulb neurons with inhalation and exhalation. Of interest, olfactory sensory neurons in the cyclic nucleotide-gated channel (CNGA2) knockout mice lose mechanosensitivity, and olfactory bulb activity does not synchronize with respiration cycle (Grosmaitre et al., 2007).

The above discussions argue for the idea that at least some of the olfactory subsystems have an important role in detecting the timing of inhalation, exhalation, and air flow in the nasal cavity. Further experiments are needed to determine the functional role of each olfactory subsystem in detecting distinct phases of the respiration cycle. The timing of inhalation and exhalation plays a pivotal role in information processing in the central olfactory system (Mori et al., 2013; Nagayama et al., 2014). Thus, our understanding of the dynamics of information processing in the large-scale networks of the central olfactory system is critically dependent on a better understanding of neuronal mechanisms for detecting respiratory phases, and for sending breath cycle signals to specific glomeruli in the olfactory bulb.

## Conflict of interest statement

The authors declare that the research was conducted in the absence of any commercial or financial relationships that could be construed as a potential conflict of interest.
